# 

**Published:** 1888-05

**Authors:** 


*Entered at the Post Office at Austin, Texas, as Second-Class Mail Matter.*

Vol. III. MAY, 1888. No. 11.

### DANIEL'S TEXAS MEDICAL JOURNAL

Subscription, $2.00 a Year, in Advance. — Single Copies, Twenty-five Cents.

F. E. DANIEL, M. D., Editor and Publisher, AUSTIN, TEXAS.

**Northern Office, 1217 Filbert Street, Philadelphia.**

